# What do emergency department pharmacist practitioners know and understand about patient safeguarding? A qualitative study

**DOI:** 10.1007/s11096-023-01663-0

**Published:** 2023-12-19

**Authors:** Daniel Greenwood, Douglas Steinke, Sandra Martin, Gary Norton, Mary P. Tully

**Affiliations:** 1 Division of Pharmacy and Optometry, University of Manchester, Oxford Road, Manchester, M13 9PT UK; 2 Division of Nursing, Midwifery and Social Work, University of Manchester, Oxford Road, Manchester, M13 9PL UK; 3 Present Address: School of Healthcare, College of Life Sciences, University of Leicester, Leicester, LE1 7RH UK; 4 Present Address: School of Pharmacy and Medical Sciences, University of Bradford, Richmond Road, Bradford, BD7 1DP UK

**Keywords:** Education, Emergency service, Hospital, Maltreatment, Pharmacists, Safeguarding

## Abstract

**Background:**

Pharmacists with additional clinical skills now work in UK emergency departments. Known as Emergency Department Pharmacist Practitioners, the role was developed in response to a shortage of physicians and nurses. They carry out activities typical of traditional hospital pharmacists, but also novel ‘practitioner’ activities such as examining patients, and acting as designated care provider. The role includes a responsibility to safeguard patients from harm. Professional competence, i.e. to safeguard patients, is underpinned by knowledge of the subject, but also knowledge application.

**Aim:**

To investigate what Emergency Department Pharmacist Practitioners know and understand about safeguarding vulnerable children and adults.

**Method:**

Thirteen Emergency Department Pharmacist Practitioners were interviewed to explore their knowledge and understanding of safeguarding. Interview questions were developed from review of relevant literature, as were vignettes with variables identified and altered to produce different scenarios. Template analysis was used to code data to a priori themes for each of the stages of the initial safeguarding process, and new themes that emerged throughout the process.

**Results:**

Six themes were identified in addition to the four a priori themes. Overall, participants frequently described how safeguarding concerns are both recognised and responded to, but seemed more comfortable when responding to medicines related concerns. Factors thought to influence the safeguarding process included: resources and setting; and education, training and experiential learning; and culture.

**Conclusion:**

While Emergency Department Pharmacist Practitioners interviewed were aware of the safeguarding process, there were some misconceptions as to the roles of different healthcare workers in this process.

**Supplementary Information:**

The online version contains supplementary material available at 10.1007/s11096-023-01663-0.

## Impact statements

- ED pharmacist practitioners have the knowledge and understanding required to safeguard patients, but training should include further insights as to the roles of different healthcare workers to ensure patient safety.
- To ensure patient protection, safeguarding training for ED pharmacists should include further training focused on documentation of concerns, relevant IT systems and the role of ‘gut instinct’ in issue recognition.
- There are discrepancies as to whether medication errors should be reported via error or safeguarding systems, which needs further investigation and clarification to ensure patients are protected.

## Introduction

Emergency departments (EDs) provide immediate care for those with urgent or life-threatening conditions [1]. Since 2013/14, EDs in the United Kingdom have failed to meet “the 4-h standard”, which requires 95% of patients are seen, treated and either discharged or admitted to hospital within 4 h [2, 3]. One reason for performance decline is a shortage of physicians and nurses which is not unique to the UK and has been described as an ‘international crisis’ [4, 5].

To counter staff shortages in UK EDs, in 2015, NHS England commissioned the University of Manchester to deliver ‘Advanced Specialist Training in Emergency Medicine’ (ASTEM) for pharmacists [6]. The 12-month programme provided training in how to independently manage and prescribe medicines for ED patients. Similar training was also commissioned in other regions and provided by other universities e.g. Aston University [7]. Described as ‘Emergency Department Pharmacist Practitioners’ (EDPPs), the ENDPAPER study concluded that these pharmacists provide both traditional pharmaceutical care (e.g. check prescriptions for clinical appropriateness), but also novel ‘practitioner’ care which may include patient management and responsibility for patients as ‘designated care provider’ [8, 9]. A role typically undertaken by ED physicians and nurse practitioners, hospitals may also allow EDPPs this responsibility as part of a multidisciplinary team and under consultant supervision. The role has also been titled ‘Pharmacist Advanced Clinical Practitioner’ [10] or ‘Advanced Clinical Practitioner Pharmacist’ [11]. Although ED pharmacist roles are common in other countries such as the United States and Australia, this enhanced role appears unique to the UK [9, 12]. With such responsibility, UK EDPPs are not only concerned with immediate clinical issues but are also responsible for their patients’ safety and wellbeing. Many patients who have suffered abuse or neglect seek medical attention at the ED [13, 14] and could thus be cared for by EDPPs.

The maltreatment of children and vulnerable adults is commonplace. Every year in the UK, approximately 10% of children who present to the ED are victims of abuse [15, 16]. In a global study of those aged 60 or over, a fifth of patients were found to have been neglected [17]. These patients require safeguarding, which means to “protect people’s health, wellbeing and human rights, and enable people to live free from harm, abuse and neglect” [18]. In healthcare practice, the initial safeguarding process comprises four stages: recognition; ensuring patient safety; documentation; and escalation [19]. Safeguarding training comprises 6 levels which increase in complexity, and as for other healthcare professionals [20], EDPPs should know and understand what safeguarding entails. Knowledge of the safeguarding process underpins professional competence [21], and increased training has been shown to increase victim recognition [22]. Professionals also need to be able to apply their knowledge to understand phenomena and develop professional intuition [23, 24].

### Aim

To investigate what Emergency Department Pharmacist Practitioners know and understand about safeguarding children and vulnerable adults.

### Ethics approval

The study was approved by University of Manchester Research Ethics Committee 3 (REF: 060416, May 2016) and then Proportionate University Research Ethics Committee (REF: 2019-5703-9175, January 2019).

## Method

EDPPs were interviewed to investigate their knowledge and understanding of safeguarding. Those eligible worked in a UK ED and had completed additional clinical training beyond a post-graduate diploma in clinical pharmacy (typically 2-years long and taken by most UK hospital pharmacists) [9].

**Fig. 1:**
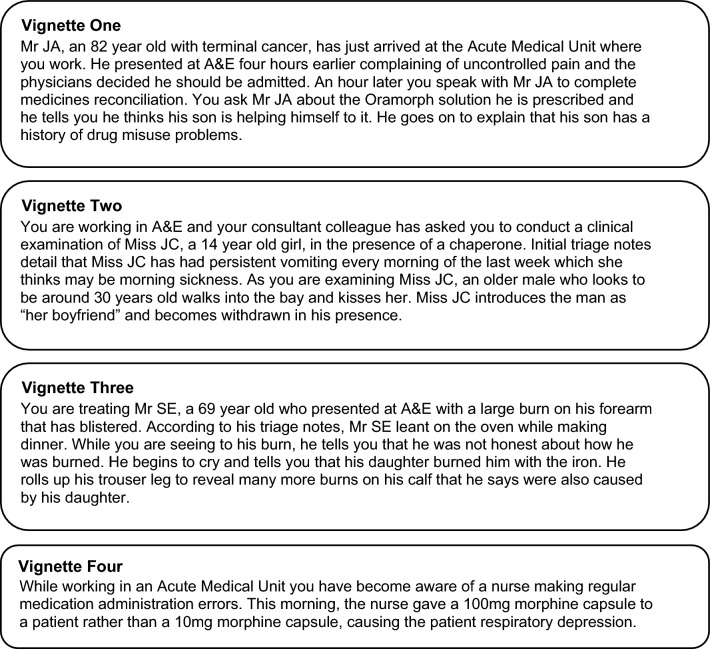
Vignettes of safeguarding situations that were used to stimulate discussion in the interviews

For recruitment, past and current ASTEM students were invited via e-mail, as were those invited to the ENDPAPER study [9]. At least 12 participants were sought to achieve thematic saturation [25]. Recruitment progressed in two phases due to competing research commitments (other funded, time-critical studies). In the first phase (2016), four EDPPs were interviewed face-to-face at their place of work. In the second phase (2019), a further nine EDPPs were interviewed by telephone and given a £25 shopping voucher for their time. Conducted by DG, interviews were semi-structured, audio-recorded and lasted an average of 40 min. The interview schedule (Supplementary material) comprised knowledge-based questions about safeguarding, but also vignettes (Fig. 1) to explore pharmacists’ understanding of the subject. Questions about the initial safeguarding process were grouped into two categories ‘recognition’ and ‘response’ so not to lead or condition participants to specific stages e.g. ‘ensure patient safety’ (Table 1).

**Table 1:** The different types of abuse and neglect suffered by children and vulnerable adults

Type of abuse/neglect	Summary
Physical	The intentional use of physical force, or implements that results in, or has the potential to result in, physical injury [40]
Psychological*	Intentional or unintentional behaviour that conveys to someone that he/she is worthless, flawed, unloved, unwanted, endangered, or valued only in meeting another’s needs [40]
Sexual	Any completed or attempted sexual act, sexual contact, or non-contact sexual interaction [40]. Non-contact sexual interaction includes indecent exposure or sexual solicitation [41, 42]
Neglect	A failure to meet someone’s basic physical, emotional, medical/dental, or educational needs; failure to provide adequate nutrition, hygiene or shelter; or failure to ensure safety [40]
Financial**	Includes theft, fraud, exploitation, pressure in connection with wills, property, inheritance or financial transactions, or the misuse or misappropriation of property, possessions or benefits [43]

*Also known as ‘emotional’ abuse

**Only vulnerable adults can be financially abused as, unlike children, they have assets [44]

Vignettes were designed through identification of variables which were altered to create different hypothetical situations (Table 2) [26]. The interview schedule was reviewed by all authors including a social worker (GN) and then pilot tested by two acute medicine pharmacists (including author SM). Four vignettes enabled exploration of varied maltreatments, balanced with interview fatigue. Informed by the ENDPAPER study [27] and feedback from pilot testing, the variable ‘EDPP activity’ was used so that two vignettes depicted ‘traditional’ activity and two depicted ‘practitioner’ activity. The variable ‘setting’ reflected anecdotal evidence that EDPPs often worked part-time in the ED, with their remaining time spent working in adjacent inpatient medical wards in ‘traditional’ roles.

**Table 2:** Variables identified and their variation for the vignettes developed

Variable	Vignette one	Vignette two	Vignette three	Vignette four
EDPP* activity	Traditional	Practitioner	Practitioner	Traditional
Setting	AMU**	ED***	ED	AMU
Type of safeguarding issue	Theft	Sexual abuse	Physical abuse	Medication error
Method of initiation	Disclosure	Disclosure	Disclosure	Recognition
Victim	Patient (elderly)	Patient (child)	Patient (elderly)	Patient(s)
Abuser/perpetrator	Victim’s son	Victim’s boyfriend	Victim’s daughter	Healthcare professional

*Emergency Department Pharmacist Practitioner

**Acute Medical Unit

***Emergency Department

Interview recordings were transcribed by a provider, with a sample of transcript excerpts checked against the recordings by DG. Template analysis was then used, with the four stages of the safeguarding process as a priori themes and further themes emerged throughout. A constant comparison process was used with previously reviewed transcripts re-reviewed as new themes emerged.

All themes were included in the qualitative synthesis, with participants′ collective knowledge and understanding of safeguarding presented. Within themes, participants′ knowledge and understanding of safeguarding were also compared. Quotes were used to explain major themes, but also more generally where these summarised participants’ collective view.

## Results

From analysis of interviews with 13 EDPPs, (7 were ASTEM students/graduates; zero drop-outs), 6 themes (4 collated under ‘General factors which influence the safeguarding process’) were identified in addition to the 4 a priori themes (collated under ‘safeguarding issue identification and response'). Saturation of themes was achieved. No differences between Phase 1&2 themes were observed, therefore all data were analysed together.

All participants had completed at least some safeguarding training but with varied extent and focus. One EDPP had Level 4 training (children) with cases referred to them; five had Level 3 (children and/or adults); three had Level 2 (children and/or adults); and four were unsure. One EDPP had Level 3 training for adults but only Level 1 for children. Two participants worked exclusively in paediatrics.

### Scope of safeguarding

Pharmacists overwhelmingly described how the aim of safeguarding is to protect patients from harm. Some took this further and emphasised patient wellbeing e.g. P5; *“it’s [safeguarding] not just about abuse, it’s also about health and wellbeing*”. In practice, safeguarding was thought of as first recognising those at risk and then raising any concerns with other care providers. Children and vulnerable adults with physical/social/mental health issues were identified as those who require safeguarding. All types of abuse were identified by participants e.g. physical and financial, but also neglect including ‘self-neglect’ where patients are unable to care for themselves.

One example of poor self-care suggested by P6 concerned older patients and their medicines:

“… elderly people not knowing what they’re supposed to be taking [medicines], maybe taking overdoses or not taking them”.

Similarly, P7 described the need to determine the mental capacity of elderly patients before counselling on medicines use:

“I wanted to provide counselling … but the patient herself would not be able to understand the information”.

Participants thought that some safeguarding concerns were more serious than others e.g. Vignette 2 which depicted potential child sexual abuse:

“A definite example of quite a serious safeguarding one [concern], you know?” (P12).

Described by P11 as ‘safeguarding queries’, concerns thought less serious but still required action included neglect due to poverty.

Various abusers were described, mostly abusive or neglectful parents, but also those who cared for adults or children in managed settings. While patient safety was a priority, the need to approach potential abusers non-accusatory was also thought important as:

“... what on the face of it might look like abuse, [that] isn’t always the case” (P3).

For example, P8 recalled how they had identified bruises on a child’s trunk and suspected abuse, which a consultant physician concluded were due to a blood disorder.

While repeat medication errors are a safeguarding issue, participants were often unsure as to whether these would be reported as a medication error or safeguarding issue. In Vignette 4, where a nurse had made repeat medication errors, most would esclate this through an error reporting system although some opted for safeguarding channels.

### Responsibility to safeguard

Pharmacists felt they had responsibilities to safeguard patients like other healthcare workers and those outside of health settings e.g. teachers. Such broad, society-wide responsibility was also acknowledged as a recent development where the ‘tolerance’ of abuse has decreased:

“I suppose as a society we’re more aware of abuse … I think [now] there’s no tolerance to abuse” (P2).

While they felt everyone had a role in safeguarding, participants thought that the specific roles of hospital workers varied.

### Safeguarding issue identification and response

Participants described all four stages of the initial safeguarding process: recognition; ensuring safety; documentation; and escalation.

#### Recognition

Generally, pharmacists were confident in their ability to recognise potential safeguarding issues i.e. develop concerns and suspicions. These were often developed when taking a patient history, performing clinical examinations or speaking with patients. The role of instinct was also described;

“... there’s a lot about instinct, which I don’t know how you train. I don’t know if that’s just an experience thing” (P2).

The need to “qualify” suspicions was thought an important first step, with the potential for false allegations acknowledged. Confirming suspicions involved asking patients about sensitive topics but also trying to correlate the injury with reported cause. Participants thought it crucial to speak to the potential victim without the suspected abuser present, to prevent coercion. Some more sensitive one-to-one clinical situations were considered good opportunities to speak with the potential victim alone e.g. diagnostic scans. With respect to patient disclosure, P6 would find it easier to respond when the disclosure is more confident and overt (e.g. Vignette 3), whereas some patient uncertainty (e.g. Vignette 1) led them to deliberate more.

Although thought an important step in confirming any suspicions, P8 felt that:

“A lot of people [professionals] don’t want to ask all of those awkward questions”

and instead would prefer to hand issues over to others. Commenting on pharmacists more generally, P6 described how:

“As a whole they [pharmacists] are more involved with the medication side… they would pass concerns on to the relevant member of staff or report it”(P6).

This was seen as a negative by P7, who had concerns that pharmacists might be *“wrapped up in our [their] own little medicines bubble”* or even be *“indifferent”* to more complex, underlying safeguarding issues. This perceived lack of ability was attributed to a lack of confidence; how other healthcare professionals see patients and raise concerns first; and those in traditional roles have limited exposure to information.

However, traditional pharmacy activities were thought advantageous to identify certain safeguarding issues, such as the covert or omitted administration of medicines:

“Lots of hidden little bits of abuse or mistreatment can manifest through medication… perhaps someone isn’t taking their medicines in the way you’d expect them to” (P2).

Participants thought that EDPPs who provide more ‘practitioner’ care would have greater involvement earlier in patient visits, and therefore be more involved with safeguarding.

Several factors were thought to impede evidence gathering and confirming suspicions. Those who cause harm were thought clever in their approach, while patients might not be forthcoming with information. The failure of professionals to document their concerns in medical notes was also thought to prohibit issue recognition, specifically because trends would be more difficult to spot. To aid with trendspotting, P2 thought it important that the hospital safeguarding team be involved with every concern as soon as possible.

The need to determine the competence of children to determine their own care was also highlighted.

Responding to Vignette 2, P7 would determine whether the 14-year-old girl involved was ‘Gillick competent’ (competent to consent to their own treatment [28]), and if so, would seek consent to share information e.g. with the safeguarding team, to ensure a legal approach.

Aside from the safeguarding team, participants also described how they would seek the support of colleagues to help confirm suspicions. For medication errors, participants would speak to those colleagues who might be responsible to try and confirm whether the error was accidental or malicious:

“... is there some clear intent here to harm patients… healthcare workers can be responsible for abuse as well” (P11).

Overall, participants were not wary of acting on concerns and saw this as their professional duty. But, they acknowledged that some issues will pass through the ED unrecognised, although less often nowadays as:

“People’s threshold is a lot lower for raising these [safeguarding issues] than it was before” (P3).

#### Ensuring safety

Patient safety was of utmost priority to participants, with issue severity directing the nature of their response e.g. for immediate safety concerns they would involve the police. Participants also described methods they would use to protect patients while in the ED, but also to prevent further issues arising. For example, having identified bruises on a child and wanting to discuss their concerns with colleagues, P8 asked a healthcare assistant to stay with the family to prevent further abuse or them leaving the ED. Another approach, P10 would admit to hospital patients who were clinically well to protect them from discharge into potential unsafe environments. The need to ensure patients clinical needs, and prevent harm, were also described:

“... because he’s obviously not receiving his drug treatment [analgesia]”(P4, Vignette 1).

Where overt communication or referral could pose a safety risk, P1 described a discreet method to inform patients of services available to them:

“You can actually give them a pack of tissues that’s got a barcode on it with the number to call” (P1).

Another perspective, participants described how potential perpetrators may also require safeguarding e.g. from drug misuse problems. Concerns about the wellbeing of nurses who make medication errors were also described by P2:

“There could be issues with this member of staff in that they’re not well themselves”.

#### Documentation of concerns

Prior to discussion of vignettes, few participants described the initial documentation of safeguarding concerns. P8 described an ED pro-forma which staff use to record any suspicions and forward to a consultant. While there were some concerns about documentation e.g. failure to record concerns about repeat ED visitors, others praised how initial safeguarding concerns are initially documented which protects patients:

“... there [was] good clear documentation that the patient could not go back to that nursing home” (P5).

Responding to vignettes, participants often described how they would document any concerns in the patient’s medical notes and simultaneously make colleagues aware verbally:

“... so I would write it in the notes, but I would also verbally discuss it with somebody”(P6,Vignette 1).

For Vignette 3, P5 would enlist a medical photographer to take pictures of injuries so that:

“Even if there is no concern in the end, at least if there is evidence of it”.

They also felt strongly about the importance of documentation:

“If it’s not documented, how do you know what’s happened? How does someone who comes to view it know it’s happened?” (P5).

#### Escalation

EDPPs escalated issues various providers within and outside of the hospital, including occupational therapists, social workers, health visitors, the police and safeguarding specialists. While some participants had experience of escalating issues and felt confident to, others described the opposite e.g. P7 whose confidence was dependent on:

“... [their] mood that day, as to how assertive you feel about approaching a situation”.

Overall, participants seemed less confident in their ability to escalate issues than to recognise them.

Particularly when responding to vignettes, participants focused on patients and how they should be central to escalation. For issues involving children, participants thought parents should be involved, and in some cases, their consent to share should be sought (e.g. where a parent has been assaulted and there might be risk to a child). Further, when informing parents, participants thought it important to be inclusive and none-abrupt, and that this should be:

“... the right time to upset the applecart [i.e. disturb the status quo]” (P11).

If not done carefully, one participant thought their own safety could be compromised if parent(s) reacted aggressively.

Aside from initial documentation, referral forms to escalate issues officially were described. Sometimes, knowing which form to complete was difficult as geographical areas did not necessarily align with local authorities. As well as healthcare workers, the importance of the police and voluntary sector was thought important in escalating issues. Where child neglect was identified and of circumstantial cause (i.e. poverty rather than malicious intent), parents were often referred to foodbanks. As they had completed Level 4 safeguarding training, P11 sometimes had less experienced staff escalate issues to them. Although they only took on this role when the hospital safeguarding team were unavailable e.g. out-of-hours, they felt this was the natural result of career progression.

Except for medication errors, most participants had limited involvement beyond formal escalation to safeguarding specialists. Again unique, P11 described longer-term involvement with a particular safeguarding case, where they gave evidence to court in a child sexual-abuse case.

### General factors thought to influence the safeguarding process

Some themes common to the entire safeguarding process were identified: resources and setting; education and training; multi-disciplinary working; and culture.

#### Resources and setting

While electronic record systems were thought useful to both monitor repeat ED visits and flag safeguarding concerns to colleagues, too many IT systems were thought to impede issue identification:

“It’s all very well recording it on one [system], but you can’t see it 2 miles down the road” (P3).

#### Education, training and experiential learning

Participants felt they needed more training to be able to safeguard effectively, with interactive, scenario-based learning, preferred. Participants felt their ability improved as they gained experience, for example with more frequent difficult conversations. Experiences were also thought important to encourage reflection, and feedback about the initial recognition and response to issues re-enforced participants confidence:

“It’s such a nice moment to have feedback on that and to know that you’re thinking about things the right way” (P12).

#### Multidisciplinary working and communication

The contribution of at least 10 different professionals was described, with involvement dependent on severity of the safeguarding concern. For more serious concerns such as child sexual abuse, greater support from senior colleagues would be sought sooner:

“I would probably want to get one of my senior colleagues in with me, so I probably wouldn’t be confident in myself that I could ask all the right questions” (P2, Vignette 2).

Despite their efforts to enlist support from other professionals and organisations, it wasn’t always straightforward. Further, communication between providers e.g. primary to secondary care and the reverse, was thought poor.

#### Culture

While not excusing particular behaviour, P8 and P11 described how patients’ cultural and religious differences should be considered as there are situations where behaviours and family norms vary according to culture:

“Sometimes the dad takes over and he says everything and the mum’s sat quietly in the corner, and that’s completely normal [in that culture]; whereas, if that was an English family… it’s not usual”(P8).

In another example, P11 suspected that their patient had been assaulted with potential cultural implications. They contacted the police who advised that *“culture basically goes out of the window”*, implying that patient safety is of utmost priority. One participant also felt that cultural differences extended to physicians, where those of different backgrounds approached potential safeguarding issues differently:

“I think, sometimes those [physicians] with an English background look at things, whereas someone with an Asian background, they don’t want to get involved with that sort of thing” (P8).

## Discussion

Overall, participants had a broad and often detailed knowledge of safeguarding. All four stages were frequently described which demonstrates EDPPs awareness of how safeguarding concerns are both recognised and responded to. Reassuringly, the society-wide change in how maltreatment is viewed and managed in response to many recent high-profile cases, was recognised. In the UK, such cases include those of Victoria Climbié [29], who in February 2000 was murdered by her great-aunt when she was 8 years old. Whether this change has been seen in other countries is unclear. A recent systematic review concluded that the COVID-19 pandemic led to a reduction in reports of global child maltreatment, but the prevalence of severe cases of maltreatment increased [30]. This only included data from 13 countries, meaning the global prevalence of maltreatment and any changes in how it is viewed and managed is unclear.

From interviews, there was a general acceptance that patients should be central to the safeguarding process and any decisions made, something advocated by many organisations which support victims of abuse and neglect [31, 32]. In particular, EDPPs recognised that self-neglect is a safeguarding concern. As 0.6% of all recorded UK ED visits concern self-harm, EDPP awareness can only prove useful in supporting those patients [33]. Recent international data suggest there has been an increase in ED visits due to self-harm, likely due to the mental health impact of the pandemic, but again data are generally limited to developed countries such the UK, USA, Canada and South Korea [34]. Greater awareness of safeguarding these patients, by pharmacists and other professionals, is arguably more pertinent than ever before.

Participants acknowledged that everyone has a role in safeguarding patients, including non-clinical healthcare workers such as cleaners whose contribution was described. Despite this, there was a suggestion that pharmacists who immediately handover concerns to their ED colleagues are ‘not really’ safeguarding, whereas the formal escalation of issues was thought of as actual safeguarding. The extent to which pharmacists might be involved with safeguarding was also attributed to differences in professional role e.g. those who manage patients are more likely to escalate issues themselves. This highlights the need to ensure pharmacists in all countries are aware of their safeguarding responsibilities, tailored to local practices. Unsurprisingly, participants were generally more comfortable when responding to medicines related concerns. In the UK, for medication errors it is currently unclear which should be escalated via safeguarding channels and this should be defined. Overall, pharmacists’ contribution to safeguarding issues which involve medicines should be exploited, as other healthcare professionals may be less familiar.

Despite the UK Royal College of Nursing suggesting that urgent and emergency care pharmacists should have Level 3 safeguarding training, some EDPPs only had Level 2 [35]. Given that increased training has been found to increase professionals’ ability to recognise maltreatment, healthcare regulators of those countries with ED pharmacist roles should ensure they are suitably trained to safeguard patients. Training should focus on the documentation of suspected maltreatment, including medical photography, as this was seldom mentioned and could be a weakness [28]. A further training focus should be how to communicate effectively with other providers, including the police, with a focus on use of relevant IT systems. Poor communication between professionals is frequently a factor which hinders safeguarding the vulnerable in the UK and other countries [29, 36–38]. A third focus for training should be the role of ‘gut instinct’ in safeguarding potential victims. Although a participant felt such instinct cannot be taught, intuition can be developed through the application of knowledge [22] and so training should incorporate interactive scenarios [39]. Scenarios should seek to stretch pharmacists’ knowledge of maltreatment and safeguarding in a simulated, and therefore safe, environment. As many participants described few opportunities to apply their knowledge in practice, training should be completed periodically to ensure continuous learning.

With respect to study limitations, author DG who led the research is a pharmacist, as are authors DS, SM and MPT. Author GN (a social worker) was involved in all stages of study design and manuscript preparation. Another limitation, 7/13 participants were students/graduates of the ASTEM programme which was delivered by the University of Manchester and led by authors DS and SM. This may limit transferability of the study given that other similar training programmes exist. To encourage authentic interview responses, the participant information sheet clearly stated that participation would have no impact whatsoever on their ASTEM progression. Recruitment in two stages, three years apart, is also a limitation as EDPP roles and safeguarding involvement could have developed over that period, however no thematic differences were identified between the groups. With regards to data coding, author DG coded data independently which prevented assessment of inter-coder reliability, but the coding template was reviewed by authors DS and MT throughout manuscript preparation.

## Conclusion

While those EDPPs interviewed were aware of the different types of maltreatment and the safeguarding process, there were some misconceptions as to the safeguarding roles and responsibilities of different healthcare workers. Safeguarding training for ED pharmacists should focus on case documentation, communication including relevant IT systems, and the role of gut instinct in the identification of maltreatment. Training should be reviewed periodically to reflect policy changes and serious cases. In the UK, where there are six advancing levels of safeguarding training, EDPPs should be trained to Level 3 as a minimum—as is recommended by the UK Royal College of Nursing. Although to be confirmed through further research, information about the recognition and escalation of medication errors via safeguarding channels should be included in safeguarding training for healthcare workers. Another topic for future research, the impact of revisions to safeguarding training on EDPPs ability to recognise and escalate safeguarding issues should be evaluated, the findings of which will indicate whether pharmacists can protect those patients entrusted to them.

### Supplementary Information

Below is the link to the electronic supplementary material.

Supplementary file1 (DOCX 18 KB)
