# Supplementary material for: What do emergency department pharmacist practitioners know and understand about patient safeguarding? A qualitative study
Source: Int J Clin Pharm. 2023 Dec 19;46(1):195–204. doi: 10.1007/s11096-023-01663-0 (PMC10830784; doi:10.1007/s11096-023-01663-0)
Supplement: Supplementary file 1 — Supplementary file1 (DOCX 18 KB) [file 11096_2023_1663_MOESM1_ESM.docx]

**Interview schedule**

**What do Emergency Department Pharmacist Practitioners know and understand about patient safeguarding?**

Daniel Greenwood^1,3^, Douglas Steinke^1^, Sandra Martin^1,3^, Gary Norton^2^, Mary P Tully^1^

^1^Division of Pharmacy and Optometry, University of Manchester, Oxford Road, Manchester, M13 9PT

^2^Division of Nursing, Midwifery and Social Work, University of Manchester, Oxford Road, Manchester, M13 9PL

^3^Present address: School of Healthcare, College of Life Sciences, University of Leicester, LE1 7RH

^4^Present address: School of Pharmacy and Medical Sciences, University of Bradford, Richmond Road, Bradford, BD7 1DP

Corresponding author: dan.greenwood@leicester.ac.uk

***Introduction***

My name is Daniel Greenwood and I am a PhD candidate at Manchester Pharmacy School evaluating Emergency Department pharmacists who have completed additional clinical skills training. Thank you for offering to participate in the final study of my PhD which is titled:

‘With a new role comes new responsibility: what do Emergency Department pharmacists who have completed additional clinical skills training know and understand about safeguarding?’

*Prior to working through the questions below, an initial discussion will take place in order to build rapport with participants. This discussion will likely involve talking about their experiences of undertaking additional clinical skills training.*

***Background details of participant***

1. Where do you currently work and what is your role?
2. Since qualifying as a pharmacist, where have you worked and in what role(s)?
3. Have you had any previous safeguarding training?

***Data collection***

1. What do you understand safeguarding to be?
2. Whose responsibility do you think safeguarding is?

What role do you think pharmacists have / should have?

1. How confident are you in your ability to identify a safeguarding issue?
2. How confident are you in your ability to respond to a safeguarding issue?
3. Have you ever identified safeguarding issues?

If yes, how did it feel to identify these issues?

If yes, how did you respond to these issues?

If yes, how did it feel to respond to these issues?

1. Have you ever been wary of responding to safeguarding issues?

If yes, what made you feel this way?

1. Four written scenarios to investigate participants understanding of safeguarding

**Vignette 1**

Mr JA, an 82 year old with terminal cancer, has just arrived at the Acute Medical Unit where you work. He presented at A&E four hours earlier complaining of uncontrolled pain and the doctors decided he should be admitted. An hour later you speak with Mr JA to complete medicines reconciliation. You ask Mr JA about the Oramorph solution he is prescribed and he tells you he thinks his son is helping himself to it. He goes on to explain that his son has a history of drug misuse problems.

*How would you approach this scenario?*

*How difficult would it be for you to decide what to do?*

*How difficult would it be for you to do what you decide to do?*

**Vignette 2**

You are working in A&E and your consultant colleague has asked you to conduct a clinical examination of Miss JC, a 14 year old girl, in the presence of a chaperone. Initial triage notes detail that Miss JC has had persistent vomiting every morning of the last week which she thinks may be morning sickness. As you are examining Miss JC, an older male who looks to be around 30 years old walks into the bay and kisses her. Miss JC introduces the man as “her boyfriend” and becomes withdrawn in his presence.

*How would you approach this scenario?*

*How difficult would it be for you to decide what to do?*

*How difficult would it be for you to do what you decide to do?*

**Vignette 3**

You are treating Mr SE, a 69 year old who presented at A&E with a large burn on his forearm that has blistered. According to his triage notes, Mr SE leant on the oven whilst making dinner. While you are seeing to his burn, he tells you that he was not honest about how he was burned. He begins to cry and tells you that his daughter burned him with the iron. He rolls up his trouser leg to reveal many more burns on his calf that he says were also caused by his daughter.

*How would you approach this scenario?*

*How difficult would it be for you to decide what to do?*

*How difficult would it be for you to do what you decide to do?*

**Vignette 4**

Whilst working in an Acute Medical Unit you have become aware of a nurse making regular medication administration errors. This morning, the nurse gave a 100mg morphine capsule to a patient rather than a 10mg morphine capsule, causing the patient respiratory depression.

*How would you approach this scenario?*

*How difficult would it be for you to decide what to do?*

*How difficult would it be for you to do what you decide to do?*

*8.* Do you think that safeguarding issues go unidentified?

If yes, why do you think this is?

If no, why do you think this is?

9. Do you think that safeguarding issues are managed appropriately?

If yes, what is good about how they are managed?

If no, what could be improved?

10. Do you have any other comments regarding safeguarding and pharmacists?

***Closing***

Thank you for your participation.
